# Adverse Cardiac Remodelling after Acute Myocardial Infarction: Old and New Biomarkers

**DOI:** 10.1155/2020/1215802

**Published:** 2020-06-12

**Authors:** Alexander E. Berezin, Alexander A. Berezin

**Affiliations:** ^1^Internal Medicine Department, State Medical University, Ministry of Health of Ukraine, Zaporozhye 69035, Ukraine; ^2^Internal Medicine Department, Medical Academy of Post-Graduate Education, Ministry of Health of Ukraine, Zaporozhye 69096, Ukraine

## Abstract

The prevalence of heart failure (HF) due to cardiac remodelling after acute myocardial infarction (AMI) does not decrease regardless of implementation of new technologies supporting opening culprit coronary artery and solving of ischemia-relating stenosis with primary percutaneous coronary intervention (PCI). Numerous studies have examined the diagnostic and prognostic potencies of circulating cardiac biomarkers in acute coronary syndrome/AMI and heart failure after AMI, and even fewer have depicted the utility of biomarkers in AMI patients undergoing primary PCI. Although complete revascularization at early period of acute coronary syndrome/AMI is an established factor for improved short-term and long-term prognosis and lowered risk of cardiovascular (CV) complications, late adverse cardiac remodelling may be a major risk factor for one-year mortality and postponded heart failure manifestation after PCI with subsequent blood flow resolving in culprit coronary artery. The aim of the review was to focus an attention on circulating biomarker as a promising tool to stratify AMI patients at high risk of poor cardiac recovery and developing HF after successful PCI. The main consideration affects biomarkers of inflammation, biomechanical myocardial stress, cardiac injury and necrosis, fibrosis, endothelial dysfunction, and vascular reparation. Clinical utilities and predictive modalities of natriuretic peptides, cardiac troponins, galectin 3, soluble suppressor tumorogenicity-2, high-sensitive C-reactive protein, growth differential factor-15, midregional proadrenomedullin, noncoding RNAs, and other biomarkers for adverse cardiac remodelling are discussed in the review.

## 1. Introduction

Heart failure (HF) is a global health problem with serious economic burden that has been considered as the dominant cause of cardiovascular (CV) morbidity and mortality in the developed and developing countries [1, 2]. HF affects around 26 million people worldwide (including 5.7 million and 3.4 million people in the US and in the EU, respectively), and the estimated expenditures for HF care were around $31 billion [1]. It is expected that by 2030 more than 40 million people will have this condition, and the HF diagnosis and therapy will increase twice and even more [3]. The clinical outcomes remain poor with a five-year survival rate of approximately 50% regardless of phenotype of HF that completely correspond to the expected survival rate in nonmetastatic cancer [3, 4]. Despite sufficient improvements in diagnosis, prevention and treatment of HF new incidences of HF with reduced ejection fraction (HFrEF) and midrange ejection fraction (HFmrEF) in contrast to HF with preserved ejection fraction (HFpEF) continue to occur as a need for heart transplantation and mechanical support device use [4]. Additionally, increased prevalence of HFpEF represents the most frequent cause of CV and sudden death, primary hospitalization, and readmission to the hospital due to acute decompensation of HF [5].

The most common primary causes of HFrEF/HFmrEF remain acute ST-segment elevation myocardial infarction (STEMI) and hypertension, while incidences of HFpEF were rather associated with hypertension, acute non-ST-segment elevation myocardial infarction (non-STEMI), and alternative causes (atrial fibrillation, cardiomyopathy, myocarditis, valvular heart disease, and diabetes mellitus) compared with STEMI [6–8]. Contemporary strategy for the prevention of HF after acute STEMI is based on early complete cardiac revascularization and prevention of negative impact of comorbidities, such as diabetes mellitus, abdominal obesity, hypertension, thyroid dysfunction, kidney failure, and conventional CV risk factors (smoking, dyslipidaemia, insulin resistance, and hyperuricemia) [9, 10]. In fact, complete recovering of blood flow through culprit coronary artery and other ischemia-related stenosis with primary percutaneous coronary intervention (PCI) is not warranted for full prevention of late adverse cardiac remodelling [11, 12]. Although improvement of prognosis, increase in quality of life, and delay in progression or reversal of ischemia-induced cardiac remodelling and chronic HF remain prime targets for the treatment of AMI [13, 14], there are no clear approaches for risk stratification in AMI patients after successful PCI [15]. For instance, hyperemic microcirculatory resistance and no-reflow phenomenon were found as strong predictors for the extent of infarct size and early cardiac remodelling [16]. Additionally, optic coherent tomography or intravascular ultrasound performed over 3 months after initial major cardiac event frequently allows identifying several factors contributing advance in late cardiac remodelling, such as silent restenosis, progression of old stenotic lesions, late stent thrombosis, and several post-PCI technical problems with incomplete stent branches' expansion, stent malposition, and underpressed culprit plaque [17, 18]. Except for early revascularization, cardiac remodelling could be prevented by pharmacotherapy including complex neurohormonal blockade and device-based therapies, which are addressed in the improvement of ventricular dyssynchrony and prevention from fatal arrhythmias [19]. In this context, new diagnostic and predictive options are needed to prevent cardiac remodelling and HF. The aim of the review was focused on the circulating biomarker as a tool to stratify postmyocardial infarction patients at high risk of poor cardiac recovery after reperfusion with primary PCI and developing HF.

## 2. Adverse Cardiac Remodelling after Acute Myocardial Infarction: Definitions and Contributing Factors

### 2.1. Definition of Adverse Cardiac Remodelling

Adverse cardiac remodelling after AMI is defined as complex interactions between cellular and extracellular components of myocardium, which are neurohumoral and epigenetic regulations, leading to changes in the cardiac architectonics and geometry frequently affecting both ventricles and atrials, worsening diastolic filling and systolic function and associated with developing heart failure [17]. Additionally, there is a large number of definitions of cardiac remodelling after STEMI, which are based on multiple imaging modalities, such as presentation of akinesia area, left ventricle enlargement, reduced LVEF, and early diastolic dysfunction (including longitudinal strain increase, twist of LV apex, and tethering effect). In fact, an impact of passive mechanical constraint of surrounding myocardium on infarct zone mediates infarct expansion and decline in both regional and global systolic function [20].

Other criteria of cardiac remodelling affect shaping stunned and hibernated myocardium after incomplete revascularization or delay of PCI performing with inadequate perfusion recovery [21]. However, non-STEMI is also associated with cardiac remodelling, rather mild-to-moderate than severe, and frequently nondistinguished from STEMI-induced cardiac disorders in prognostic aspects, but the canonic model of pathogenesis of adverse cardiac remodelling was based on STEMI impact on cardiac architectonic. There is a sustainable option that STEMI-induced cardiac remodelling frequently relates to HFrEF/HFmrEF, but non-STEMI-induced cardiac remodelling is rather associated with developing HFpEF than HFrEF.

### 2.2. Contributing Factors of Adverse Cardiac Remodelling

**Figure 1:**
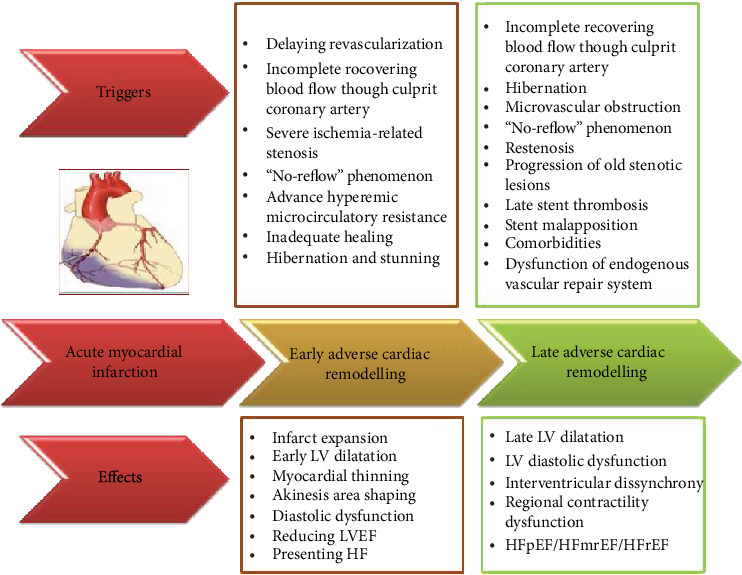
Adverse cardiac remodelling after AMI: the role of different triggers in development of cardiac architectonic disorders and heart failure. LV: left ventricular; HF: heart failure; HFpEF: HF with preserved ejection fraction; HFmrEF: HF with midrange ejection fraction; HFrEF: HF with reduced ejection fraction.

In fact, there are at least two different variants of adverse cardiac remodelling after acute MI, which distinguished each other in pathogenesis, so called the early (at 2-3 weeks after initial event) and late (at 3-6 months after AMI) remodelling (see Figure 1).

Contemporary point of view is based on an idea that early complete primary revascularization of culprit artery and ischemia-induced stenosis/occlusions in other coronary arteries at first hours of STEMI is independent and the most powerful factor preventing early LV cavity dilation, declining LV pump function and the developing of HF. It has been postulated that preserved systolic function and LV dimensions at early stage of various revascularization procedures can accompany with myocardial biomechanical and energetics stress, mitochondrial dysfunction, and oxidative stress that lead to potent fatal arrhythmias even prior to diastolic dysfunction developing [22]. Over the next three months after restoring TIMI III blood flow through culprit artery with PCI, the primary causes inducing adverse cardiac remodelling can be different from the aforementioned. Indeed, other factors that may contribute to cardiac remodelling after successful primary PCI are arterial healing, vessel remodelling, stent restenosis, thrombosis, and incomplete expansion of stent branches (known as malposition), and stent fracture, which require ischemia-driven target vessel revascularization further [23]. Performing of optical coherence tomography (OCT) in STEMI patients presenting with late and very late stent thrombosis has yielded that stent malposition was determined in 55% cases, quarter of which had been found evidence of positive vessel remodelling [24]. Additionally, neoatherosclerosis and uncovered stent struts were reported as the primary cause of late thrombosis in 35% cases and 10% cases, respectively [24]. Although coronary stent fracture is an underrecognized event, it has been reported frequently in the drug-eluting stent era [25]. However, investigators have shown that technical problems with first-generation eluting stent implantations in STEMI patients were associated with higher in-hospital mortality and posthospital target vessel failure or cardiac death [24].

Endothelial shear stress, neointima formation, and late thrombosis can appear beyond inadequate PCI and stent positioning and are result of accelerating atherosclerosis and inadequate drug support, i.e., nonoptimal care with statins, refusal from dual antiplatelet therapy, effective anticoagulation if needed, and adenosine intracoronary for prevention no-reflow/slow-flow phenomena. Even a novel device (known as bioabsorbable cardiac matrix) was not able to attenuate adverse cardiac remodelling after AMI [26], while there were strong positive expectations regarding these devices [27]. Despite implantation of second-generation everolimus-eluting stent in STEMI appears to be better to first-generation eluting stents, there is evidence that even a small degree of chronic intrastent conditions may significantly influence on healing persistence [28]. Frequencies of uncovered and malapposed struts as well as percentage of stents fully covered with neointima were 1.2%, 0.4%, and 60.9%, respectively, for over a one-year period after PCI with second-generation everolimus-eluting stent implantation [28]. In fact, they were not associated with the incidence of clinical events and intrastent thrombus.

The next factor contributing to early and late cardiac remodelling is the “no-reflow” phenomenon. Indeed, the “no-reflow” phenomenon can be considered as a component of early cardiac remodelling after STEMI that relates to microvascular obstruction and dysfunction causing severe disturbance in regional perfusion [29]. In fact, the “no-reflow” phenomenon is a result in poor healing of the culprit artery and adverse cardiac remodelling, increasing the risk for major adverse cardiac events, such as recurrent MI, newly diagnosed HF, and sudden death, but the “slow-flow” phenomenon appears to be a serious factor contributing to both types of adverse cardiac remodelling [30, 31].

Additional factor that is involved onto a development of late adverse remodelling is epigenetically mediating disturbance of endogenous vascular repair system [32, 33]. It has been found that altered vascular repair has maintained vasoconstriction and vascular dysfunction that accelerated atherosclerosis and supported hibernation in the grey zone around myocardial infarction. Overall, the development of adverse cardiac remodelling after AMI regardless of initial cause (even in asymptomatic patients) was consistently associated with poor clinical outcomes, and it could be predicted and completely resolved [34, 35].

**Figure 2:**
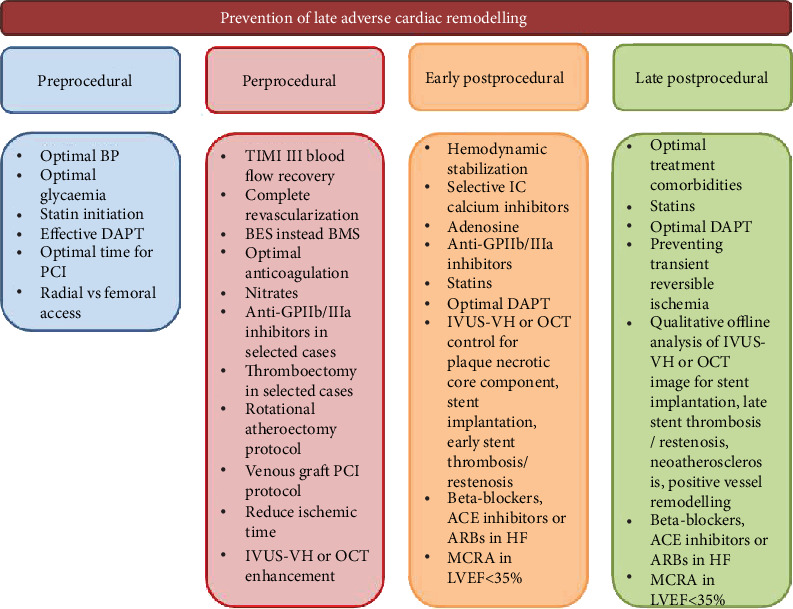
The factors preventing late adverse cardiac remodelling in AMI patients after successful reperfusion with PCI. IVUS-VH: intravascular ultrasound virtual-histology; BMS: bare metal stent; BES: biolimus eluting stent; OCT: optical coherence tomography; DAPT: dual antiplatelet therapy; ACE: angiotensin-converting enzyme; ARBs: angiotensin-II receptor antagonists; MCRA: mineralocorticoid receptor antagonists; IC: intracoronary.

The factors preventing late adverse cardiac remodelling after successful reperfusion with primary PCI in STEMI patients are indicated in Figure 2. Recognition of the heterogeneous pathophysiology of adverse cardiac remodelling after AMI can create a powerful risk stratification score based on biomarkers reflecting various stages of pathogenesis of the condition [36].

## 3. Pathogenetic Mechanisms of Adverse Cardiac Remodelling after Acute Myocardial Infarction

**Figure 3:**
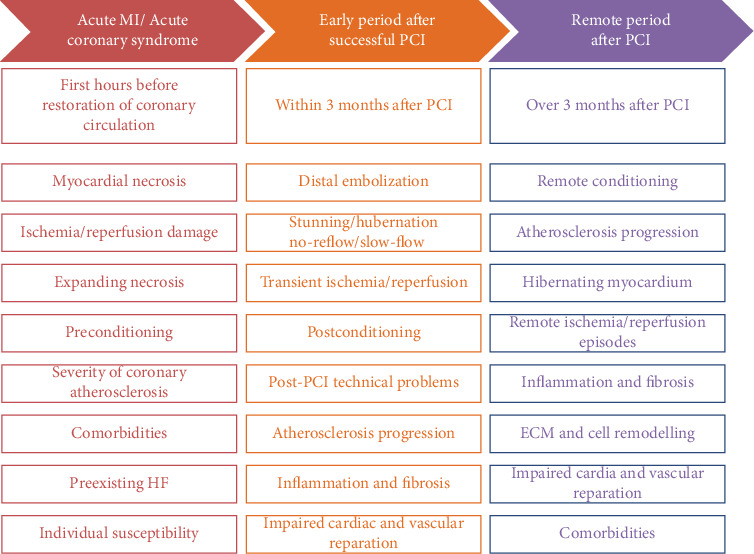
The main pathogenetic mechanisms underlying the initiation and progression of late adverse cardiac remodelling in AMI patients after successful reperfusion with PCI. HF: heart failure; ECM: extracellular matrix; PCI: percutaneous coronary intervention.

Advances in our understanding of the molecular mechanisms of regulation toward late adverse cardiac remodelling were associated with the breakthrough in the recognition of interplaying between various processes translating ischemia/reperfusion injury on myocardium, such as disrupting nitric oxide (NO) and vascular endothelial growth factor (VEGF) signalling systems, p38 MAPK pathway and redox dysregulation, cytokine release, and activation of apoptotic and necrotic death pathways with subsequent stimulation of oxidative stress, mitochondrial dysfunction, altered myocardial cell metabolism, excessive fibrosis, and cardiac cell remodelling [37]. Therefore, preserved microvascular inflammation, small vessel obstruction, endothelial dysfunction, and atherosclerotic lesions mediate a remote effect on advance LV remodelling [38]. Additionally, there are new explanations regarding individual susceptibility to ischemia/reperfusion injury including early and remote ischemic preconditioning [39].  Figure 3 yields main pathogenetic mechanisms that are involved in the pathogenesis of late adverse cardiac remodelling.

In fact, restoration of adequate blood perfusion after a critical period of ischemia and prevention of reperfusion damage appear to be not the only protector over cardiac damage. Early irreversible cardiac myocyte injury leading to necrosis in the ischemic myocardium and expanding infarction zone are an attribute of susceptibility of cardiac cells to impaired metabolism, loss of structural integrity and selective permeability of the cell membranes, altered ultrastructure of cell organoids, such as sarcolemma disruption, deterioration of nucleus, ribosomes, mitochondria, and sarcoplasmic reticulum, the presentation of mitochondrial amorphous densities, and chromatin fragmentation [40]. During this early stage of AMI development, the mitochondrial dysfunction plays a pivotal role in cardiac myocyte apoptosis in the ischemic/reperfused heart, cardiac necrosis, and ischemia-induced preconditioning phenomenon [41, 42].

Numerous studies have shown that proapoptotic stimuli through involving cytokines, which belong to the B-cell lymphoma 2 (Bcl-2) super family, mediate the permeability of the mitochondrial membranes and stimulate the release of a wide spectrum of the active apoptogenic molecules (cytochrome c, Bax) into the cytoplasm. They cause the apoptotic response, peroxidation of membrane, and disruption of mitochondrial chromatin materials, including small interfering ribonucleic acid (RNA) and mitochondrial deoxyribonucleic acid (DNA) [43, 44]. Cytochrome C is able to bind to the adaptor protein apoptotic protease activating factor 1 (Apaf-1) and act as a trigger of its oligomerization that activates caspase cascade through initiating procaspase-9 recruitment. Caspases including caspase-6 and caspase-9 cleave cellular proteins and DNAs/RNAs emerging apoptosis [45]. This process is under the close epigenetic regulation of long noncoding RNAs (LncRNA) and microRNAs (miRNA-29b-1-5p, miRNA-195), which negatively regulate Bcl2l2 gene expression and participate in cardiac myocyte apoptosis, oxidative stress through inducing hydrogen peroxide (H_2_O_2_), and inflammation via triggering proinflammatory cytokine release [44, 46]. Therefore, downregulated miRNA-98 and miRNA-124 may attenuate cell survival through diminished levels of STAT3 and p-STAT3 in response to ischemia and over production of H_2_O_2_ [47, 48]. During ischemia/reperfusion episodes, oxidative stress, mitochondrial Ca^2+^ overload, proinflammatory cytokines (interleukin- (IL-) 2, IL-6, tumor necrosis factor-alpha, and interferon-gamma) stimulate the activity of the matrix metalloproteinases and suppress release of their tissue inhibitors [49]. MMPs (MMP-2, MMP-6, and MMP-9) directly contribute to global and local myocardial contractile dysfunction and induce cell death [50]. Other matricellular proteins, such as thrombospondin- (TSP-) 1 and TSP-2, as well as bone-related proteins (osteopontin, osteonectin, and osteoprotegerin), were found to regulate cardiac reparation and remodelling via activation of VEGF and transforming growth factor (TGF-*β*) by binding to the latency-associated propeptide, inhibition of MMP activity, and exertion of potent angiostatic actions of antigen-presenting cells and T-cells [51]. Moreover, they are triggers for accumulation, degradation and remodelling of extracellular matrix (ECM), cleaving big endothelin-1 and attenuating vasoconstriction, and modification of architectonics of myocardium leading to cardiac remodelling and HF development [51, 52].

Interestingly, susceptibility of myocardium to ischemia and reperfusion may relate to various inhered causes, such as mutations in genes encoding for angiotensin II, angiotensin-converting enzyme, osteopontin, osteoprotegerin, CC chemokine receptor 2, the members of the family of multidomain extracellular protease enzymes ADAMTS (A Disintegrin and Metalloproteinase with Thrombospondin motifs), predominantly ADAMTS-2, ADAMTS-4, ADAMTS-10, and ADAMTS-13, promoter region of endothelial NO synthase, apelin, TGF-*β*, VEGF, galectin-3, ficolin-1, S100 calcium-binding protein A9, and mitochondrial aldehyde dehydrogenase 2 (NDUFC2) [53–56]. Finally, susceptibility of cardiac cells to ischemia and reperfusion damage may relate to the capability of endogenous redox systems to protect cell membranes and cellular structures (mitochondria, cytoskeletal proteins, growth factor receptors, and microtubule-associated proteins) from the impaired effect of the deteriorating energetic metabolism and detergenting impact of oxidized lipids and proteins sustaining an effective work of transmembrane ionic pumps [57]. This phenomenon was called ischemic preconditioning, and now it is also recognized as an early (before AMI or during acute phase of AMI) and remote (overreparative period of AMI) phenomenon depending on a period of onset of ischemia-reperfusion episodes. However, previous studies have revealed a reduction of infarct size and peripheral area with hibernating/stunning myocardium with both types of preconditioning due to intracardiac protection that prevents cytosolic and mitochondrial Ca^2+^ overload, accumulation of reactive oxygen species (ROS), lysosomal/nonlysosomal enzyme releasing, and inflammatory reaction [58–60].

Recurrent episodes of ischemia/reperfusion induce cardioprotective mechanisms in failed heart named postconditioning and remote conditioning [61, 62], which are supported by various comorbidities (diabetes mellitus, insulin resistance, obesity, and inflammatory conditions) [63, 64]. The cardiac protective mechanisms may include upregulation of caveolin, resolvin D1/E1, ubiquinone, long pentraxin PTX3, apelin, glucocorticoids, and long noncoding RNAs expression for IL-19, VEGF, eNO synthase, haem oxygenase-1 (HO-1), calcitonin gene-related peptide, and peroxisome proliferator-activated receptor gamma, and downregulation of *β*-adrenergic signalling, G protein-coupled receptor kinase-2, and *β*-arrestin 1 and 2 in cardiac myocytes, fibroblasts/myofibroblasts, tissue residence cells, and circulating progenitor cells as well as mononuclears [65–68]. These factors reduce inflammatory infiltrates, stabilize cell membrane, support membrane ionic channels, and suppress the formation of key proinflammatory cytokines, such as tumor necrosis factor-alpha (TNF-*α*), IL-1*β*, and IL-6. Additionally, IL-19 suppresses the polarization of proinflammatory subtype M1 macrophages and triggers M2 macrophage polarization in infarct myocardium that leads to inhibition of cardiac remodelling [68].

During AMI and recurrent episodes of micronecrosis in myocardium after PCI due to remote ischemia/reperfusion damage, the important role in the regulation of cardiac remodelling belongs to alarmins, which are released by necrotic myocardium and act as a powerful trigger of inflammatory cytokine synthesis [69]. Damaged and necrotic cardiac myocytes secrete wide-spectrum factors called DAMPs (Damage-Associated Molecular Patterns), such as high-mobility group 1B protein (HMGB1), RNA, nucleotides, heat shock proteins (HSP), members of the S100 family, and IL-1a, which potentiate the inflammatory response, attenuate oxidative stress, act as direct cytotoxic agents, and induce thrombus formation and circulating blood cell aggregation [70]. Numerous molecules, such as HMGB1, S100 family members, are able to induce apoptosis of circulating endothelial progenitor cells and tissue residence cells via a multiligand receptor for advanced glycation end products- (RAGE-) mediated activation of endoplasmic reticulum stress pathway [71]. Therefore, the DAMPs and other chemokines, such as CXC and CC (predominantly CCL2, CCR2, CCR5, and ELR+CXC chemokines), recruit various subpopulations of peripheral blood cells including proinflammatory mononuclears, regulatory T-cells, nature killers, and neutrophils in the infarcted myocardium and endothelium supporting inflammatory response [72].

Inflammation is a crucial element for clearance of cellular and matrix debris, while suppression of proinflammatory signalling is necessary to transform the inflammatory phase to the proliferative phase [73]. Indeed, proinflammatory mediators include uncoupling protein 2, superoxide dismutase- (SOD-) 1 and SOD-2, ROS, through the activation of mTOR, hypoxia-induced factor- (HIF-) 1, Toll-like receptor (TLR)/IL-1, and RAGE-dependent pathways in surviving border-zone fibroblasts, cardiac myocytes, endothelial cells, smooth muscle cells, mononuclears, and several residence and progenitor cells mediating reparative processes [74–76]. It relates to the modification of cardiac fibroblasts into myofibroblasts that are enriched in *α*-smooth muscle actin, accumulation of extracellular matrix, neovascularization, and angiogenesis. However, the proinflammatory cytokines may have a detrimental impact on cardiac remodelling and function directly maintaining repetitive ischemia/reperfusion episodes, suppressing reparation, supporting endothelial dysfunction, coagulation, and thrombosis [77–79].

Over a 3-month period after PCI, the extracellular matrix is continually being remodelled, and tissue fibroblasts, myofibroblasts, and antigen-presenting cells become quiescent and undergo apoptosis, and cell debris is cleared by macrophages [80–82]. The regulation of proliferative response and changing of cellular phases of tissue inflammation are mediated by the renin-angiotensin-aldosterone system (RAAS) and simpatico adrenal system, which also are central players in the endogenous repair system [83, 84]. Additionally, the autonomic nervous system may play a crucial role in the inflammatory and apoptotic remodelling following AMI [85]. Thus, late adverse cardiac remodelling is a sophisticated structural and functional response of failing heart to numerous triggers (inflammation, fibrosis, cell survival signalling, and *β*-adrenergic signalling) and damaged factors (ischemia, reperfusion, necrosis, and apoptosis) that appear consequently and mutually activate each other.

## 4. Biomarkers of Adverse Cardiac Remodelling

Although there are well-developed current clinical recommendations for HF provided by the experts of the European Society of Cardiology (2016) [86] and American College of Cardiology/American Heart Association (2017) [87], there is a lack of statements for the use of biomarker strategies for diagnosis, prediction, stratification, and prevention of adverse cardiac remodelling. In fact, cardiac remodelling after AMI regardless of PCI and other approaches for revascularization is strongly associated with the development and progress of HF. In this context, early biomarkers of myocardial injury and necrosis, as well as biomarkers of biomechanical stress, neurohumoral and inflammatory activation, and fibrosis, having predictive and diagnostic evidence for acute and chronic HF, are extrapolated over strategy regarding diagnosis, outcomes, and stratification of adverse cardiac remodelling (Table 1).

There is no complete agreement between experts from the European Society of Cardiology and American College of Cardiology/American Heart Association regarding the utility of biomarkers in HF [88]. Natriuretic peptides (NPs) are recommended by both guidelines for acute and chronic HF diagnosis, prediction of HF-relating outcomes, including death, and a risk stratification. In contrast, the European Society of Cardiology (2016) HF clinical recommendation does not consist the supporting evidence regarding other biomarkers for multitask strategy in HF, and HF-guided therapy is not routinely recommended, while the HF biomarker guidance was previously approved by the American College of Cardiology/American Heart Association. Additionally, there was poor discrimination when NPs were used in patients with HF at hospital discharge, which was inferior to its performance in patients with ambulatory HF regardless of severity cardiac dysfunction and phenotypes.

However, there is a large body of evidence that other biomarkers (growth/differential factor-15, MMP-2, MMP-6, MMP-9, adipocytokines (apelin, chemerin, and visfatin), circulating endothelial and mononuclear progenitor cells, activated and apoptotic endothelial cell-derived microvesicles, miRNAs, and bone-related proteins) reflecting different stages of the pathogenesis of adverse cardiac remodelling after PCI can be considered as promising tools for further strategies to improve prediction of clinical outcomes, attenuate CV risk stratification, and develop personifying strategy for treatment [89, 90]. For instance, miRNAs are speculated to have crucial roles in the nature evolution of adverse cardiac remodelling after AMI, and identification of key genes associated with damaged heart response could improve prediction models for the patients [91]. Moreover, miRNA profiling and gene cards with information about a signature of mutations involved in the regulation of the transcription factors, which mediate cardiac remodelling, appear to be promising for further precise medicine after PCI [92].

## 5. Biomarkers of Cardiac Injury and Necrosis

Elevated levels of high-specific cardiac troponins T (hs-TnT) and I (hs-TnI) in peripheral blood are served as diagnostic and predictive biomarkers for acute coronary syndromes and AMI [93], as well as an independent prognosticator of CV risk in the general population [94]. Cardiac troponins are structure proteins of actin-myosin complex, which are released from the cells due to necrosis or leakage from cytosol through the permeable cell membrane [95]. High-sensitivity cardiac troponin assay allows diagnosing patients with minor myocardial injury and suggesting a size of infarction [96]. Cell-free pool of cardiac troponins was reported having a tendency to decrease after AMI, while peak concentrations of both hs-TnT and hs-TnI have strongly predicted major cardiovascular events including death, recurrent MI, need of PCI, and subsequent HF hospitalization [96, 97]. Moreover, elevated concentrations of circulating cardiac troponins remain useful independent predictive biomarkers of newly post-AMI HF [98, 99]. Interestingly, elevated levels of hs-TnI were associated with CV death, whereas hs-TnT has more strongly predicted the risk of non-CV death [100]. In fact, cardiac and noncardiac surgeries mediate the elevation of troponins in the peripheral blood postprocedurally. It requires specific approach to assay an impact of transient elevation of these findings on a risk of poor prognosis. Obviously, the combined biomarkers' models are necessary.

After a prolonged period of hopes regarding improvement of diagnostic and risk stratification in STEMI patients with subsequent PCI using the combined biomarker models (cardiac troponins, NPs, copeptin, choline, soluble ST2, GDF-15, high-sensitivity C-reactive protein, galectin-3, and lipoprotein-associated phospholipase A2) [101], it has clearly become what large clinical trials need to evaluate diagnostic and predictive values of various combinations of biomarkers, because the evidence of previous studies in AMI patient treated with PCI appeared to be controversial [102, 103]. Copeptin did not add diagnostic information to peak concentration of high-sensitive troponin T in STEMI patients with subsequent PCI [104, 105]. Yet, hs-TnT/hs-TnI and NT-probrain NP (NT-proBNP) were recognized to have similar predictive values for all-cause mortality and first readmission in HFpEF [106, 107], whereas NT-proBNP was superior to cardiac troponins for the prognostication of HFrEF clinical outcomes [108, 109]. It has been noted that the predictive value of hs-TnI for HF-related clinical outcome was strongest in men with HFpEF/HFrEF than in women [108]. Other biomarkers, including soluble ST2, high-sensitivity C-reactive protein, galectin-3, midregional proadrenomedullin, and GDF-15, in combination with hs-TnI/hs-TnT did not represent superiority in comparison with the isolated use of hs-TnI/hs-TnT in HFpEF, whereas in patients with HFmrEF/HFrEF, multimarkers' strategy was better in the prognostication of poor prognosis [110, 111].

Although previous clinical trials did not find significant interactions between stable HFpEF and HFrEF when considering the prognostic value of the NT-proBNP, cystatin-C, hs-TnT, and soluble ST2 [112–114], it can be otherwise for HF that is associated with adverse cardiac remodelling after AMI with subsequent PCI. Thus, clinical prediction models for HF-related outcomes based on various biomarkers of biomechanical stress (NT-proBNP, copeptin, midregional proadrenomedullin (MR-proADM), and growth/differential factor- (GDF-) 15), inflammation (high-sensitivity C-reactive protein), and fibrosis (galectin-3, soluble ST2) were only improved marginally by the addition of hs-TnT/hs-TnI. Moreover, hs-TnT or hs-TnI added to NT-proBNP and sST2 appears to be emerging biomarkers in the prediction of adverse outcome of HF after AMI in a short-term period [115], but whether this combination is most suitable for remote prognostication in patients with known late adverse cardiac remodelling and different phenotypes of ischemia-induced HF is not fully clear.

## 6. Inflammatory Biomarkers

### 6.1. Interleukins

IL-1*β*, IL-6, and angiopoietin-like protein 2 (Angptl 2) are inflammatory cytokines that influence deleterious effects on myocardium structure and function unleashing to cardiac remodelling [116]. There is strong evidence clarifying that the myocardial expression levels of IL-1*β*, IL-6, and Angptl 2 were significantly higher in the AMI patients than in the healthy volunteers [117]. Moreover, the levels of Angptl 2 and IL-6 rather correlated with the severity of coronary atherosclerosis than the size of the infarct area and HF presence. In contrast, IL-1*β* levels were associated with prior HF admissions, functional cardiac impairment, and higher NT-proBNP, sST2, and hs-TnT concentrations [115]. In fact, circulating IL-1*β* levels had been clinically meaningful in HF patients interfering with the predictive ability of sST2. Indeed, regardless of LVEF, HF patients with low sST2 (≥35.0 ng/ml) and also low IL-1*β* (≥49.1 pg/ml) had significantly lower risk of CV death, HF-related outcomes including readmission, than among patients with high sST2 (>35.0 ng/ml) and also high IL-1*β* (<49.1 pg/ml) levels [115].

### 6.2. Soluble Suppression of Tumorigenicity-2

Serum levels of IL-33 and soluble suppression of tumorigenicity-2 (sST2), which is the soluble form of IL-1 receptor-like 1 (IL-33), were significantly higher in HF regardless of the presence HF phenotypes associated with HF symptom severity, LV hypertrophy, and the risk of CV death and hospitalization than in healthy volunteers [118, 119]. It was found that IL-33 improved cell viability after ischemia injury through ST2 signalling and suppression nuclear factor kappa-B that unleashed the upexpression of the antiapoptotic factors (XIAP, cIAP1, surviving) and HIF-1, preventing apoptosis [120]. In patients with AMI, serum levels of sST2 were found to be increased, and after adjustment for comorbidities, the Killip class and troponin T sST2 independently predicted the excess risk of death and HF [121]. Development of adverse cardiac remodelling due to AMI was strongly associated with the elevated levels of sST2 in the peripheral blood [122].

Serum sST2 served as a predictive biomarker in patients at risk of HF and in individuals with established chronic HF [123], but the prognostic value of the biomarker was diminished after adjusting for the clinical status including comorbidity presence (abdominal obesity, diabetes mellitus, and obstructive pulmonary disease) and NT-proBNP [124–126]. Additionally, sST2 was able to be helpful in short-term clinical outcome prognostication in acute HF and actually decompensated HF patients regardless of worsening kidney function, whereas renal failure was found to be a crucial factor for the NP predictive value [127, 128]. In-patients survived after acute HF have yielded the concentrations of sST2 at discharge which were independently associated with sudden death, CV death, HF-related death, and HF readmission during the 3-month period after discharge [127, 128]. Yet, sST2 yielded strong, independent predictive value for all-cause and cardiovascular mortality, and HF hospitalization in chronic HF, and deserves consideration to be part of a multimarker panel together with NT-proBNP and hs-TnT [129]. The PARADIGM-HF trial (Prospective Comparison of ARNI With ACEI to Determine Impact on Global Mortality and Morbidity in Heart Failure) has revealed the levels of sST2 increased at 1 month which were associated with worse subsequent HF clinical outcomes, and the decreased sST2 concentrations were related to better prognosis particularly related to declined CV death and HF admission [130].

### 6.3. C-Reactive Protein

High-sensitive C-reactive protein (hs-CRP) has also markedly improved the risk stratification of acute HF and acutely decompensated HF patients in multibiomarker models, which predominantly included MR-proADM and NT-proBNP [131, 132]. However, circulating levels of hs-CRP were associated with the New York Heart Association functional class of HF, primary hospitalizations and readmission predominantly in patients with HFrEF, but not HFpEF [133]. Unfortunately, hs-CRP did not add incremental value to NPs, sST2, and galectin-3 in patients with HFpEF rather than HFpEF [133, 134]. The ASCEND-HF trial has reported that the levels of hs-CRP at admission in acute HF patients were not associated with acute dyspnea improvement, in-hospital death, advancing HF, short-term (30 days) and long-term (180 days) mortality, and HF readmission [135, 136]. On the contrary, at 30 days, elevated levels of hs-CRP among survivors were associated with higher 180-day mortality and readmission [135]. Although hs-CRP is under ongoing investigations, potential treatment options and goals of the therapy among HF individuals are not fully determined.

### 6.4. Growth Differential Factor-15

Growth differentiation factor- (GDF-) 15 is determined as an inflammation and oxidative stress biomarker, which belongs to the TGF-*β* cytokine superfamily and is highly expressed in myocardium and endothelial cells in CV disease including HF [137]. Previous studies have shown that GDF-15 protected the myocardium from ischemia and reperfusion injury [138, 139]. Higher serum levels of GDF-15 were associated with poor prognosis in acute HF independent from concentrations of NPs [140] and chronic HF irrespective of LVEF [141, 142]. Moreover, the Valsartan Heart Failure Trial has shown that serial measurements of GDF-15 had increased the incremental predictive power to the only measure at baseline for the severity of HF and prognosis [143]. Additionally, the elevated serum level of GDF-15 was the most prognostic biomarker in comparison to NT-proBNP, hs-CRP, and hs-TnT, in predicting long-term mortality in advanced HF [144]. Overall, a multimarker model based on NT-proBNP, hs-CRP, GDF-15, and hs-TnT had more predictable HFrEF and HFpEF than the isolating biomarker [145, 146]. Probably, inflammatory mediators, such as sST2 and GDF-15, as it is expecting, can become molecular targets not only for the diagnosis but also for the treatment of adverse cardiac remodelling in the future.

## 7. Biomarkers of Cardiac Fibrosis

### 7.1. Galectin-3

Over the last decade, galectin-3 had been widely investigated as a biomarker of fibrosis and inflammation with a promising predictive value for HF development and CV events [147]. Galectin-3 is multifunction *β*-galactoside-binding protein, which belongs to lectin family and is expressed in several tissues and circulating cells, such as mononuclears, macrophages, progenitor cells, mast cells, and neutrophils [148]. Galectin-3 plays a pivotal role in inflammation, fibrosis, immunity, tissue repair, and cardiac remodelling and acts as a mediator of the development and progression of the diseases, for which these pathogenetic stages are crucial [149, 150]. Indeed, galectin-3 is expressed in myocardium releasing from activated macrophages and contributes cardiac dysfunction through the remodelling of ECM and accumulation of collagen [151]. Additionally, galectin-3 is able to mediate cardiac and vascular fibrosis induced by overexpressed aldosterone [152]. However, there is evidence confirming the role of polymorphism of galectin-3 gene in susceptibility to cardiac injury and fibrosis [153]. Being a mediator of both mutual relating processes—inflammation and fibrosis—galectin-3 was approved by the Food and Drugs Administration (USA) as a predictive biomarker for HF development and progression [87, 154]. In fact, elevated levels of galectin-3 were found in patients with adverse cardiac remodelling regardless of HF phenotypes and it ethnologies [155, 156]. Therefore, galectin-3 having some advantages to NPs (more stability and resistance against hemodynamic overload and unloading state) predicted CV mortality and rehospitalization in HFrEF and HFpEF [157, 158]. Moreover, the TRIUMPH (Translational Initiative on Unique and Novel Strategies for Management of Patients with Heart Failure) has shown that repeated measures of serum levels of galectin-3 could be useful in routine clinical practice for HF prognostication and treatment monitoring [159]. However, head-to-head comparison of sST2 and galectin3 has revealed the superiority of sST2 in long-term risk stratification in an ambulatory stable HF [160]. For future direction, these facts require to be investigated in detail in large clinical trials with large sample size, because a meta-analysis of a discriminative value of galectin-3 did not yield a confirmation of previously received data [161].

### 7.2. Biomarkers of Collagen Turnover

It has been postulated that biomarkers of collagen turnover, such as carboxy-terminal telopeptide of collagen type I, amino-terminal propeptide of type III procollagen, MMPs, and tissue inhibitors of MMPs, may be useful for risk stratification of cardiac remodelling associated with HFpEF and HFrEF [162, 163]. Indeed, myocardial fibrosis being a major cause of diastolic dysfunction contributes predominantly to the HFpEF [164]. The ECM rearrangement corresponds to an intensity of the inflammation in myocardium, and serum levels of biomarkers of collagen turnover are mediated by a balance between degradation of ECM components and synthesis. Proliferative phase complimented to myocardial fibrosis is considered a typical response during late adverse cardiac remodelling, whereas increased degradation of ECM is suitable for AMI and early cardiac dilatation [165]. In fact, MMP-2, MMP-9, carboxytelopeptides of procollagen type I, and aminopeptide of procollagen type III had a predictive value for HFpEF that was equal NT-proBNP [163], while discriminative ability of elevated serum levels of MMP-2 was superior to NT-proBNP for early HFpEF [162, 163, 166]. Whether emerging biomarkers of ECM rearrangement and collagen turnover is essential to identify asymptomatic patients with HFpEF after AMI with subsequent PCI is not fully clear, while a loss of myocardial collagen scaffolding plays a pivotal role in adverse cardiac remodelling with poor prognosis. Interestingly, elevated levels of C-terminal telopeptide were associated with global LVEF, the risk of CV death, and newly diagnosed or worsening HF due to various causes [167–169]. In this context, integrity of ECM biomarkers into personifying predictive strategy in AMI patients appears to be promised, because multiple biomarkers' approach with traditional biomarkers and indicators of ECM turnover may have increased the sensitivity and specificity of clinical outcomes in patients with adverse cardiac remodelling and isolated diastolic dysfunction.

## 8. Biomarkers of Biomechanical Myocardial Stress

### 8.1. Natriuretic Peptides

The physiologically natriuretic peptide (NP) system mediates water and sodium homeostasis playing a pivotal role in blood pressure enhancement, fluid retention, vascular function, structure remodelling of the heart, kidney, and vessels, and maintaining differentiation and repair tissue, and supports immunity, metabolic response, and inflammation [170]. There are at least four members of NP system, such as atrial NP (ANP), brain NP (BNP), C-type of NP, and D-type of NP [171]. Biological effects of NPs are provided through interacting with appropriate receptors: NPR-A, NPR-B, and NPR-C. Kidney effects of NPs are diuresis and wateresis due to the decreasing tubular reabsorption of sodium and water, increasing glomerular filtration rate (GFR) in result of inducing afferent arteriole vasodilation, and protection of the kidney from metabolic and ischemia injury [172]. Vascular effects of NPs correspond to vasodilation, support, capillary permeability and vascular reparation, and antiproliferative and hypocoagulative effects [173]. NPs mediate cardiac protection with respect to decreasing preload and afterload, diminishing biomechanical stress, and maintaining anti-ischemic, antiproliferative, and antiapoptotic abilities. Therefore, NPs have direct inotropic and antiarrhythmic effects [174]. Overall, the NP system is a physiological antagonist of RAAS and the sympathoadrenal system. The main triggers for synthesis and release of NPs are myocardial stretching, fluid retention, increase of pre- and postload, BP elevation, decreasing GFR, and ischemia of target organs (kidney, heart, and brain). Therefore, adipocytes and glial cells can produce NPs as a result of proinflammatory stimulation [175].

Increased activity of a circulating and local NP system was determined in patients with CV disease including LV hypertrophy, AMI, stable coronary artery disease, hypertension, and HF [176]. However, there are large numbers of causes distinguishing from CV and accompanying elevation of circulating levels of NPs (see Table 2). There is a large body of evidence showing that NP production occurs in close relation to the severity of LV systolic dysfunction, and the circulating levels of BNP and ANP strongly correspond to the New York Heart Association functional class of HF [88]. However, the production of NPs in advanced HF became blunt and irrespective of how high concentration of NPs in peripheral blood fluid retention, vasoconstriction, and cardiac dysfunction appears to progressed. In contrast, adequate treatment of HF, which is associated with improvement of clinical status and increase of tolerance to physical exercise, corresponds to declining circulating levels of BNP and ANP [177].

Therefore, patients with abdominal obesity frequently present less levels of BNP that it is expected due to increased circulating levels of neprilysin, which degradates BNP [178]. Although older age and female sex are the most common reason association with increased levels of NPs in circulation beyond relative causes, some structural abnormalities corresponding to decreased mean *e*′ velocity and increased mitral early flow velocity/early diastolic tissue velocity ratio can be found [179–181].

Current clinical recommendations are considered NPs predominantly BNP, NT-proBNP, and NT-proANP, as diagnostic and predictive biomarkers for HF regardless of LVEF, as well as a tool for risk stratification in general population [86, 87]. However, elevated levels of NPs (BNP ≥ 100 pg/ml or NT − proBNP ≥ 300 pg/ml; or BNP ≥ 300 pg/ml or NT − proBNP ≥ 900 pg/ml if in atrial fibrillation/flutter) in patients with suspected HFmrEF/HFpEF were found to confirm the diagnosis [182]. NPs are also excellent prognostic biomarkers of adverse cardiac remodelling after AMI, whereas the clinical value of such discriminative ability is less clear than established acute and chronic HF [183]. Therefore, decreased levels of NT − proBNP < 1000 pg/ml as a result of HF therapy was associated with lower 180-day mortality and readmission in comparison with NT − proBNP ≥ 1000 pg/ml, whereas NT-proBNP reduction of >30% from initial levels did not improve 6-month outcomes and was not more effective than a traditional treatment [184–186]. Overall, elevated levels of NPs including NT-proBNP and NT-proANP had higher negative diagnostic value than the positive diagnostic value for HF, while the positive predictive ability of NPs in elevating concentrations was superior to the negative predictive value for asymptomatic cardiac remodelling, as well as HF regardless of LVEF. In fact, high individual variability, depending on the serum levels of NPs on comorbidities, including GFR, abdominal obesity, and older age and female sex, gives more opportunities to rule out major structural cardiac abnormalities and HF, when NP levels are normal or near normal. Confirmation of the HF and cardiac remodelling with isolating diastolic dysfunction requires more predictive information including clinical conditions, diastolic characteristics, measure of LVEF, and other biomarker assay.

### 8.2. Copeptin

Copeptin is a stable 39-aminoacid glycopeptide derived from C-terminal portion of the precursor of arginine vasopressin, which is a key regulator of water homeostasis and plasma osmolality [187]. Serum levels of copeptin have exhibited close linear correlation with concentrations of arginine vasopressin and are use as surrogate biomarker of its secretion [188]. There is evidence that elevated serum levels of copeptin are a diagnostic biomarker of asymptomatic cardiac remodelling, HF, sepsis, acute kidney injury, insulin resistance, and metabolic syndrome [189]. Several trials have yielded that increased levels of copeptin were strong predictor of mortality in patients with acute and chronic HF [189, 190], stroke [191], end stage of renal disease [192], stable CAD [193], and diabetes mellitus [194]. However, there is a large number of confounding factors (hydration status, gender, blood pressure, GFR, and body mass), which make it difficult to interpret data of copeptin levels in patients with known CV disease, as well as in healthy individuals [195]. Additionally, copeptin was not better than the NPs in the diagnosis and prognosis of HF as well as in prognostication of adverse cardiac remodelling after AMI [196].

### 8.3. Midregional Proadrenomedullin

Midregional proadrenomedullin (MR-proADM) is stable peptide fragment that is precursor for adrenomedullin (ADM) and generated through posttranslational processing from pre-proadrenomedullin [197]. ADM is expressed in several tissues (adrenal medulla, brain, kidney, lung, spleen, liver, and vasculature) and cells (endothelial cells, cardiac myocytes, vascular smooth muscle cells, and epithelial cells) and mediates natriuresis, diuresis, vasodilation, positive inotropic effect, and hypotension [198].

Early clinical trials have shown that circulating levels of MR-proADM were significantly increased in patients with acute HF and STEMI [199, 200], and a cut-off value of 0.79 nmol/l has been yielded to be associated with adverse outcomes including death [201, 202]. Additionally, serum levels of MR-proADM >0.70 nmol/l were proposed to be the rule-in criteria of AMI [203].

The MR-proADM has become a biomarker that was specifically investigated as a possible prognosticator of acute HF and early outcomes in STEMI patients undergoing PCI. The BACH (Biomarkers in Acute Heart Failure) study revealed that increased serum levels of MR-proANP were a useful diagnostic biomarker as BNP for acute HF in patients with acute dyspnoe [204]. The results of the DANAMI-3 (The Danish Study of Optimal Acute Treatment of Patients with ST-segment-elevation myocardial infarction) study have shown that elevated levels of MR-proADM were strong predictor of short- and long-term mortality and hospital admission for HF after AMI [205]. Unfortunately, MR-proADM has demonstrated predictive ability with high similarity to BNP, MR-proANP, and copeptin for one-year all-cause mortality in acute HF [206]. However, the measure of MR-proADM may give additional diagnostic and prognostic information for incident CV events associated with advanced atherosclerosis that is useful for risk stratification among patients with adverse cardiac remodelling after AMI with subsequent PCI [207, 208]. Therefore, MR-proADM was able to predict major adverse cardiac events in patients suspecting AMI regardless of HF [209]. Moreover, in contrast to NPs, MR-proADM did not exhibit lowered concentration in obese patients with known HF that may facilitate diagnosis and prognosis of HF in this patient population [210].

## 9. Other Biomarkers of Cardiac Remodelling

### 9.1. Noncoding RNAs

**Figure 4:**
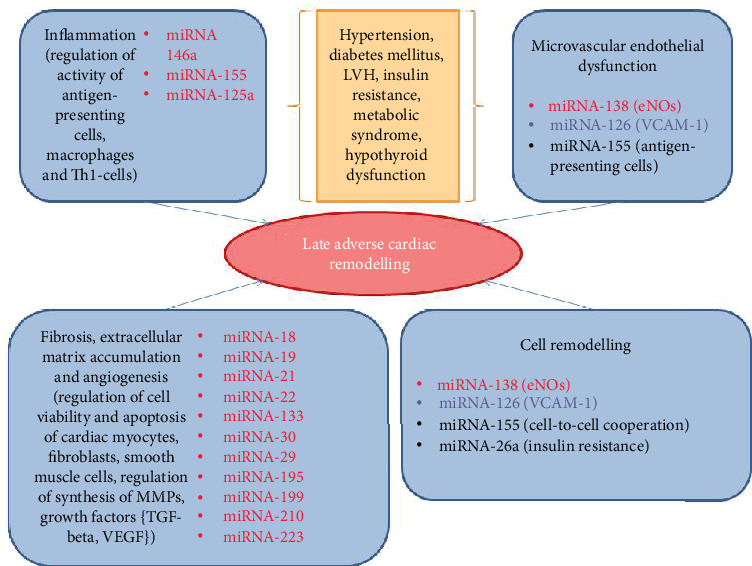
The role of miRNAs in the pathogenesis of late adverse cardiac remodelling in AMI. VEGF: vascular endothelial growth factor; TGF: transforming growth factor; NO: nitric oxide; eNOs: endothelial NO synthase; MMP: matrix metalloproteinase; VCAM: vascular adhesive molecule.

Noncoding RNAs are powerful epigenetic regulators of cardiac gene expression and mediators of cardiac homeostasis and functions [211]. There are several types of noncoding RNAs, such as microRNAs (miRNAs), long noncoding RNAs, and circular RNAs, which play a central role in the regulation of numerous pathogenetic mechanisms and coordinate coupling of morbidity state with susceptibility to inflammatory and proliferative response [212]. Among these types of noncoding RNAs, various miRNAs are widely investigated (see Figure 4). Although there is a large body of evidence regarding up- and downregulation of genes for potassium channels, SERCA, subunits of receptors, signal molecules, proinflammatory cytokines, apoptotic mediators (Bax, caspase-9) in myocardium [213–216], and miRNAs are considered rather targets for personifying intervention and translational therapy, as well as prognosticators than diagnostic biomarkers for adverse cardiac remodelling and HF [217]. However, having signatures of miRNAs, which correspond to adverse cardiac remodelling, HF, sudden death, and cardiac abnormalities with established poor prognosis, such as concentric LV hypertrophy, fibrosis, and inflammation, it has not completely understood whether the “miRNA card” personally created for each patient will have clinical significance in the prediction of HF [33, 218].

### 9.2. Circulating Mononuclear and Endothelial Progenitor Cells

Mononuclears (MPCs) and endothelial progenitor cells (EPCs) are essential components of endogenous vascular repair system that is activated as a result of several triggers, such as ischemia/hypoxia, inflammation, shear stress, thrombosis, infiltration of lipids, direct injury of vasculature, and endothelium [32].

It has been hypothesized that mobilization of MPCs/EPCs and increase in growth and differentiation into mature cells in vasculature accompany acute events, including AMI and acute HF, and are associated with vascular reparation [219, 220]. However, previous acute CV events and chronic metabolic and CV diseases, such as diabetes mellitus, abdominal obesity, and hypertension, were reported to be causes of an exhausting pool of circulating angiopoetic MPCs/EPCs with immune phenotypes CD45+CD34+, CD45+CD34+CD133+, and CD45+CD34+CD133+CD184+ [221]. Consequently, advanced HF and progression of AMI-induced adverse cardiac remodelling were related to impaired vascular repair, vascularization, and angiogenesis due to a declined number of circulating precursors and lowered their function and survival [222]. This phenomenon is known as progenitor cell dysfunction and considered a promising predictive biomarker for CV mortality and HF progression and admission [223, 224], as well as in patient population with AMI submitted to PCI [225]. Probably, coronary circulating proangiogenic MPCs/EPCs collected from coronary sinus in AMI patients with subsequent PCI can become a powerful biomarker with increased accuracy in the prediction of adverse cardiac remodelling. However, the number and functionality of proangiogenic circulating precursors appear as promising biomarkers for the prediction of cardiac remodelling and HF development. Large clinical trials are required to clearly understand the role of new biomarkers in the diagnostic and predictive strategies among AMI patients with PCI.

### 9.3. Future Perspectives

There are numerous biomarkers, which were investigated as candidates for risk stratification and prognosis in AMI patients with PCI, such as activated and apoptotic endothelial cell-derived microvesicles, bone-related proteins (osteopontin, osteoprotegerin, and osteonectin), adipokines, gastrointestinal hormones, apelin, cardiotrophin-1, defensin-1 and defensin-2, macrophage inhibitory cytokine-1, circular RNAs, and gene card. Although the data received appear to be promising, there is no clear understanding whether diagnostic and predictive abilities of these biomarkers will be better than the conventional biomarkers of biomechanical stress, inflammation, fibrosis, and cardiac injury.

## 10. Conclusions

Circulating biomarkers are a promising tool to stratify AMI patients undergoing PCI at high risk of poor cardiac recovery and HF development. NPs are traditionally recommended as diagnostic and predictive biomarkers for acute HF and chronic HF regardless of LVEF, whereas sST2, galectin-3, and cardiac troponins can be used optionally. Previous clinical studies have yielded that multimarker models, which were based on the combination of biomarkers of several pathological axes involved in the nature evolution of adverse cardiac remodelling (biomechanical myocardial stress, necrosis and injury of cardiac myocytes, and inflammation), have provided incremental prognostic information for prediction of CV death or HF in AMI patients with subsequent PCI. Future clinical trials with larger sample sizes are required to elucidate the role of personifying biomarker-based strategy for diagnostic, prediction, and treatment among patients suspecting adverse cardiac remodelling and HF.

## Figures and Tables

**Table 1:** Clinical relevance of circulating biomarkers for late adverse cardiac remodelling: overlap with HF.

Biomarkers	Heart failure				Adverse cardiac remodelling		
	Diagnosis	Outcomes	Guided therapy	Risk stratification	Diagnosis	Outcomes	Risk stratification
Currently used or recommended biomarkers							
hs-troponin T/I^҂^	-	++	--	+	+	+	+
NPs^#҂^	++	+++	+	++	++	+++	++
MR-proADM	+	+++	-	++	+	+++	++
Galectin-3^҂^	-	+	-	+	-	++	+
sST2^҂^	--	++	+	-	-	+++	+
Promising biomarkers							
Copeptin	+	++	-	+	+	++	+
GDF15	--	++	-	+	-	++	++
hs-CRP	--	+	-	-	--	+	+
IL-1*β*	--	+	-	+	--	+	+
IL-6	--	+	-	+	--	+	+
MMP-2	--	+	--	--	+	+	+
MMP-9	--	+	--	--	+	+	+
CTPpC-I	--	+	--	+	-	+	++
APpC-III	--	+	--	+	-	+	++
miRNAs	--	+	+	+	-	+	+

^−^Mildly disagree; ^--^moderately disagree; ^+^mildly agree; ^++^moderately agree; ^+++^strongly agree; ^#^approved by the European Society of Cardiology (2016); ^҂^approved by the American College of Cardiology/American Heart Association (2017). hs: high sensitive; HF: heart failure; NPs: natriuretic peptides; sST2: soluble suppression of tumorigenicity-2; MR-proADM: midregional proadrenomedullin; GDF: growth/differential factor; CRP: C-reactive protein; miRNAs: microribonucleic acids; MMP: matrix metalloproteinase; CTPpC-I: carboxytelopeptides of procollagen type I; APpC-III: aminopeptide of procollagen type III.

**Table 2:** CV and non-CV causes of elevating NPs in peripheral blood.

CV causes	Non-CV causes
Acute and chronic HF	Sepsis/shock
LV hypertrophy	Severe infections
Pulmonary hypertension	Critical ill patients
ACS/AMI	Acute and chronic kidney failure
Stable CAD	Severe trauma/surgery
Multifocal atherosclerosis	Chronic obstructive pulmonary disease
Cardiomyopathies	Severe bronchial asthma
Myocarditis	Pneumonia
Atrial fibrillation and flutter	Large burns and frostbite
Hypertension	Stroke
Congenital and acquired valvular heart disease	Kidney amyloidosis
Pericardial disease	Diabetes mellitus
Cardiac toxicity due to tumoricidal therapy	Thyroid dysfunction
Electrical cardioversion/ablation	Anemia
Successful resuscitation	Pleural disease

HF: heart failure; LV: left ventricular; ACS: acute coronary syndrome; AMI: acute myocardial infarction; CAD: coronary artery disease.

## Data Availability

This is a narrative review, so dataset was not created.

## Abbreviations

ACE: Angiotensin-converting enzyme
ADAMTS: A Disintegrin and Metalloproteinase with Thrombospondin motifs
ADM: adrenomedullin
AMI: Acute myocardial infarction
Apaf-1: Adaptor protein apoptotic protease activating factor 1
ARBs: Angiotensin-II receptor antagonists
Bcl-2: B-cell lymphoma 2
BES: Biolimus eluting stent
BMS: Bare metal stent
CRP: C-reactive protein
CV: Cardiovascular
DAMPs: Damage-Associated Molecular Patterns
DAPT: Dual antiplatelet therapy
DNA: Deoxyribonucleic acid
ECM: Extracellular matrix
EF: Ejection fraction
GDF15: Growth/differential factor-15
H_2_O_2_: Hydrogen peroxide
HF: Heart failure
HFmrEF: Heart failure with midrange ejection fraction
HFpEF: Heart failure with preserved ejection fraction
HFrEF: Heart failure with reduced ejection fraction
HIF: Hypoxia-induce factor
HMGB1: High-mobility group 1B protein
HO-1: Haem oxygenase-1
HSP: Heat shock proteins
IC: Intracoronary
IL: Interleukin
IVUS-VH: Intravascular ultrasound virtual-histology
LncRNA: Long noncoding RNA
LV: Left ventricle
MAPK: Mitogen-activated protein kinase
MCRA: Mineralocorticoid receptor antagonists
miRNA: MicroRNA
MMP: Matrix metalloproteinase
NO: Nitric oxide
NPs: Natriuretic peptides
OCT: Optical coherence tomography
PCI: Percutaneous coronary intervention
RAAS: Renin-angiotensin-aldosterone system
RNA: Ribonucleic acid
ROS: Reactive oxygen species
SOD: Superoxide dismutase
sST2: Soluble suppression of tumorigenicity-2
STEMI: ST-segment elevation myocardial infarction
TIMI score: Thrombolysis in Myocardial Infarction score
TGF: Transforming growth factor
TLR: Toll-like receptor
TNF: Tumor necrosis factor
TSP: Thrombospondin
VEGF: Vascular endothelial growth factor.
